# A comprehensive expression analysis of the expansin gene family in potato (*Solanum tuberosum*) discloses stress-responsive expansin-like B genes for drought and heat tolerances

**DOI:** 10.1371/journal.pone.0219837

**Published:** 2019-07-18

**Authors:** Yongkun Chen, Bo Zhang, Canhui Li, Chunxia Lei, Chunyan Kong, Yu Yang, Ming Gong

**Affiliations:** 1 School of Life Science, Yunnan Normal University, Kunming, China; 2 Joint Academy of Potato Science, Yunnan Normal University, Kunming, China; University of Melbourne, AUSTRALIA

## Abstract

Expansin is a type of cell wall elongation and stress relaxation protein involved in various developmental processes and stress resistances in plant. In this study, we identified 36 potato (*Solanum tuberosum* L.) genes belonging to the expansin (*StEXP*) gene family from the genome reference. These genes included 24 α-expansins (*StEXPAs*), five β-expansins (*StEXPBs*), one expansin-like A (*StEXLA*) and six expansin-like B (*StEXLBs*). The RNA-Seq analysis conducted from a variety of tissue types showed 34 expansins differentially expressed among tissues, some of which only expressed in specific tissues. Most of the *StEXPAs* and *StEXPB2* transcripts were more abundant in young tuber compared with other tissues, suggesting they likely play a role in tuber development. There were 31 genes, especially *StEXLB6*, showed differential expression under the treatments of ABA, IAA and GA3, as well as under the drought and heat stresses, indicating they were likely involved in potato stress resistance. In addition, the gene co-expression analysis indicated the *StEXLBs* likely contribute to a wider range of stress resistances compared with other genes. We found the *StEXLA* and six *StEXLBs* expressed differently under a range of abiotic stresses (salt, alkaline, heavy metals, drought, heat, and cold stresses), which likely participated in the associated signaling pathways. Comparing with the control group, potato growing under the drought or heat stresses exhibited up-regulation of the all six *StEXLB* genes in leaves, whereas, the *StEXLB3*, *StEXLB4*, *StEXLB5* and *StEXLB6* showed relatively higher expression levels in roots. This suggested these genes likely played a role in the drought and heat tolerance. Overall, this study has shown the potential role of the *StEXP* genes in potato growth and stress tolerance, and provided fundamental resources for the future studies in potato breeding.

## Introduction

Expansin, a class of pH-dependent protein family, plays a role in cell wall proliferation and growth [1,2]. Generally, it is believed that expansin bounds to glucan-coated cellulose in cell wall causing reversible disruption of hydrogen bond between cellulose microfibrils and glucan matrix, which results in cell expansion or elongation through increasing cell wall extensibility [1,3–5]. The typical expansins (containing 250–275 amino acids and two conserved domains) are divided into four subfamilies: α-expansins (EXPA), β-expansins (EXPB), expansin-like A (EXLA), and expansin-like B (EXLB) [6].

A variety of expansin genes have been identified from a range of species. Among all these genes, the functions of EXPA and EXPB have been mostly studied, which are found to be involved in multiple processes of plant development through regulating the roles of cell walls [7,8]. For example, they are found to contribute to cell wall loosening in rice coleoptile [9,10], *Arabidopsis* petiole growth [11], tomato fruit softening [12], rose petal expansion [13], soybean root system architecture [14], cotton fiber elongation [15], and tobacco leaf enlargement and internode growth [16]. Expansins are also involved in cell expansion and cell wall changes induced by phytohormones such as gibberellin (GA), abscisic acid (ABA), auxin, and ethylene, as well as biotic and abiotic stresses including heat, drought, salt and heavy metals [7,17,18]. In specific, the overexpression of rose expansin gene *RhEXPA4* in *Arabidopsis* enhances plant tolerance to drought stress, salt stress, and ABA content [19,20]. The overexpression of wheat expansin genes *TaEXPB2* and *TaEXPB23* increases the transgenic tobacco tolerance to drought [21], high salt and high temperature [22], oxidative stress [16,23,24], and water stress [25]. Some expansin genes are involved in the plant resistance to cadmium (Cd). For example, the heterologous expression of *TaEXPA2* can increase the Cd resistance of tobacco [26]. Eleven expansin genes are involved in the response to Cd stress in the Cd hyperaccumulator of *Phytolacca americana* [27]. The roles of expansin genes playing in plant development and stress-resistance have provided opportunities in plant breeding for regulating leaf size, fruit growth, root development, biotic and abiotic stress resistance, etc. [28].

Parts of expansin genes have been identified in potato (*Solanum tuberosum* L.), but still largely restricted to those genes involved in its growth and development, and abiotic stresses. Specifically, nine *StEXPA*s have been recently found to be involved in the growth and development of tubers and stems, and *StEXPA1*, *StEXPA4* and *StEXPA5* are also hormone-regulated [29]. Two *StEXP* genes (PGSC0003DMG400029331 and PGSC0003DMG400009951) homologous to the *Arabidopsis* expansin11 (AT1G20190) showed expression increase under the cold plate-treatment, whereas significant decrease under the heat [30]. Although these results have been obtained, the research on StEXP family is still very limited. Potato is the third most important food crop in the world and often suffering from drought, heat, salt and some other environmental stresses. Several reports have shown that expansins participate in resistance to these stresses [18,28]. However, it is not clear which expansins are involved in which kinds of stresses in potato.

In this study, we identified potato expansins and their corresponding genes (*StEXP*) from the genome and transcriptomes, and then analyzed their phylogenetic relationships, gene and protein structures. The expression patterns of *StEXPs* in different organs as well as under different hormone and abiotic stress treatments are studied. Quantitative real-time PCR experiment was also performed to investigate the roles of seven *StEXLs* in multiple abiotic stress, such as salt, alkaline, heavy metals, drought, heat, and cold stresses.

## Materials and methods

### Genome-wide identification of expansin proteins and genes

A total of 130 expansin amino acid sequences from *Arabidopsis thaliana*, poplar (*Populus trichocarpa*) and rice (*Oryza sativa*) were used to search sequence homologs in the potato genome published on Phytozome v12 using BLAST program (https://phytozome.jgi.doe.gov/pz/portal.html#!search?show=BLAST). Moreover, the keyword "expansin" was used to obtain expansin information from the Phytozome (https://phytozome.jgi.doe.gov/pz/portal.html#!info?alias=Org Stuberosum) and the Spud DB Potato Genomics Resource (http://solanaceae.plantbiology.msu.edu/) databases. All the target amino acid sequences were downloaded and their conserved domains were analyzed at the Conserved Domain Database (CDD) (https://www.ncbi.nlm.nih.gov/cdd) with expect value <0.05. After the repeated sequences and the sequences without pfam 03330 and pfam 01357 domains [7] were excluded from the target amino acid sequences, the remained were considered as candidate expansins. All the candidate expansins were then confirmed with online BLASTP (https://blast.ncbi.nlm.nih.gov/) and those without best hit being expansins were discarded.

### StEXPs structure, conserved domain, motif, and phylogenetic analysis

Gene structure was obtained through aligning each expansin gene coding sequence (CDS) to the genomic DNA sequences and displayed using the online Gene Structure Display Server (GSDS) 2.0 (http://gsds.cbi.pku.edu.cn/). The Multiple Expectation Maximisation for Motif elicitation (MEME) tool (http://meme-suite.org/index.html) was used to identify conserved protein domain and motif. Multiple sequence alignments of *Arabidopsis*, rice, poplar, and potato expansins were performed using ClustalW within MEGA7 [31], and then the phylogenetic tree was constructed by MEGA7 (neighbor-joining method; Poisson correction model; 1,000 bootstrap tests).

### Chromosomal localization of *StEXP*

StEXPs were mapped on potato chromosome and displayed by MapInspect software (http://mapinspect.apponic.com/) according to the potato expansin gene positions in the Spud DB database. The segmental duplicated and tandem repeated genes were determined through the ClustalW alignment comparison of all expansins with a threshold of similarity >75% and their genomic locations, and tandem duplicated genes are restricted within the range of 100 kb distance [32].

### Expression profiling of *StEXP*

The RNA-Seq data used for generating gene expression levels were downloaded from the Spud DB. These data were sequenced from many tissues of the heterozygous diploid potato (RH89-039-16 (RH)) or the doubled monoploid potato (Group Phureja clone DM1-3 (DM)) under various treatments. The sequenced tissues included tuber, root, stem, flower, petiole, stolon, tuber pith, tuber peels, and tuber cortex, and treatment condition covered 50 μmol L^-1^ abscisic acid (ABA), 10 μmol L^-1^ indole-3-acetic acid (IAA), 50 μmol L^-1^ gibberellin A3 (GA3) and 10 μmol L^-1^ 6-benzylaminopurine (BAP) for 24 h, and biotic and abiotic stresses such as 150 mmol L^-1^ NaCl, 260 μmol L^-1^ mannitol, 35°C high temperature for 24 h, and 2 days water stress, *Phytophthora infestans*, 2 mg ml^-1^ BABA (DL-β-aminobutyric), and 10 μg ml^-1^BTH (benzo (1, 2, 3)-thiadiazole-7-carbothionic acid-*S*-methyl ester) [33]. Gene expression profiling was produced using MeV v4.9 [34]. The FPKM = 0 was replaced by FPKM = 0.01 and then all the FPKM data were undergone log_2_FPKM transformation. The fold change of gene differential expression was calculated as: log_2_ (FPKM_Treatment_ / FPKM_Control_).

### Weighted gene co-expression network analysis (WGCNA) of *StEXP*

WGCNA was performed to deduce the highly co-expressed gene clusters using the WGCNA program in R package [35]. An unsigned type of topological overlap matrix (TOM) was constructed with β = 16 and then the correlation between the potato expansin genes and the selected differentially expressed genes were analyzed. The resulted co-expression network was visualized using Cytoscape 3.6.1 [36] and analyzed using Network Analyzer in Cytoscape.

### Quantitative real‑time PCR (qRT-PCR) analysis of StEXLs

The hydroponic seedlings of tetraploid potato ‘Cooperation-88’ were transplanted to Pearl Rock Medium and cultured at 25°C (16 h light/8 h dark). These seedlings were firstly irrigated by 1/4 Hoagland’s nutrient solution for three times within 15 days. Then The Pearl Rock Medium of seedlings were overflowed thrice by 1/4 Hoagland's nutrient solution containing 150 mmol L^-1^ NaCl, 10 mmol L^-1^ NaHCO_3_, 5 mmol L^-1^ ZnSO_4_, 20% PEG6000, or 1/4 Hoagland's solution, respectively. NaCl, NaHCO_3_, ZnSO_4_ and PEG6000 treated seedlings were cultured at 25°C for 24h. 1/4 Hoagland's flowed seedlings were respectively placed at 35°C, 4°C and 25°C for 24h, as the heat, low temperature stress and control. All the seedlings were given the same photoperiod (16 h light/8 h dark). The root and leaf samples were collected for qRT-PCR analysis. Total RNA was isolated from all samples using Trizol (Invitrogen, USA) method and then reverse-transcribed into cDNA using PrimeScript RT reagent Kit with gDNA Eraser (Takara, China). qRT-PCR was performed on Roche LightCycler 96 Real Time PCR System (Roche, Switzerland) with a final volume of 20 μl containing 2 μl of a 1/10 diluted cDNA template, 10 μl of the 2× TB Green Premix Ex Taq II (Takara, China) and 1.5 μl (5 mM) of gene-specific forward and reverse primers. The specific primers were designed with Primer Premier 5.0 software (PREMIER Biosoft, USA) based on the conserved part of CDS sequences, all the primer sequences used in the qRT-PCR were listed in Supplement S1 Table. The qRT-PCR program was set to a 30s preincubation at 95°C, 2 step amplification of 45 cycles at 95°C for 5s and 60°C for 5s, following a 60°C to 97°C melting curve analysis at the final step. Three independent biological repetitions and three parallel reactions were conducted in qRT-PCR. The relative expression level of target genes was analyzed using the 2^-△Ct^ method [37] with *S*. *tuberosum* elongation factor-1alpha (*EF1α*) used as the reference gene [38].

## Results

### Expansin and corresponding genes

**Fig 1:**
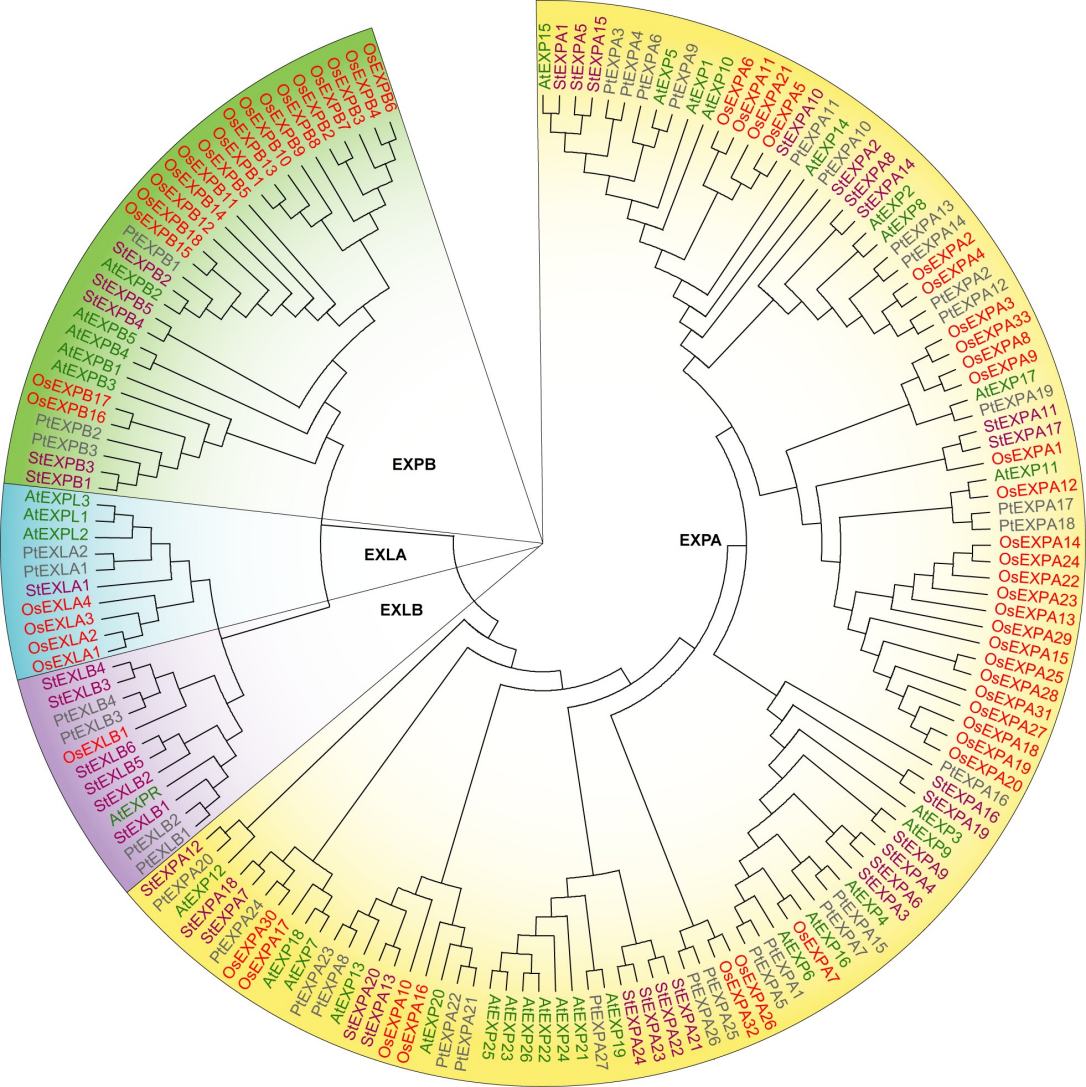
Phylogenetic analysis of expansins from *Arabidopsis*, poplar, rice, and potato. The evolutionary tree of expansin were constructed by MEGA7 software, using ClustalW alignment, Maximum Likelihood method, Equal input model, Bootstrap method, and 1,000 repetitions. The expansins in *Arabidopsis*, poplar, rice, and potato were presented in green, grey, red, and purple words, respectively.

A total of 36 candidate *StEXP*s were identified and shown in Table 1. According to the evolutionary analysis of amino acid sequences (Fig 1), 36 *StEXP* genes were divided into 4 subfamilies, *StEXPA*, *StEXPB*, *StEXLA*, and *StEXLB*, that contain 24, 5, 1 and 6 member(s), respectively (Table 1). The expansins encoded by these genes had 199–279 amino acids and their molecular weights were between 21.45 and 30.28 kD. In addition, the theoretical pI (isoelectric point) of these StEXPs proteins ranged from 4.68 to 9.87. Specifically, the pI of StEXPAs and StEXPBs (except StEXPB5) were all more than 7.0, while that of the StEXLBs (except for StEXLB2) were below 7.0. As the averaged value of hydropathicity (GRAVY) of these proteins (except for StEXPA6, StEXPA10, StEXPA18, StEXPB4, and StEXLA1) were negative, most of the StEXPs were hydrophilic proteins. The instability coefficients of these expansins were between 17.79 and 50.85 (only two expansins being more than 40), that is, most of these expansins were stable.

**Table 1:** Description of expansin's genes identified from potato genome.

Gene	Encoding amino acid no.	Molecular weight (kD)	Theoretical pI	GRAVY	Instability index	Aliphatic index
StEXPA1	241	25.96	9.49	-0.076	25.39	72.86
StEXPA2	256	28.05	9.38	-0.120	36.09	70.51
StEXPA3	259	27.99	9.43	-0.052	19.09	71.20
StEXPA4	257	28.06	9.39	-0.012	30.41	78.13
StEXPA5	239	25.54	9.36	-0.031	29.06	69.00
StEXPA6	260	28.28	9.32	0.026	36.26	72.08
StEXPA7	266	28.60	9.10	-0.029	23.60	65.34
StEXPA8	258	27.63	8.56	-0.132	23.91	68.49
StEXPA9	261	28.56	9.55	-0.013	29.59	76.25
StEXPA10	250	26.88	8.45	0.038	26.13	69.84
StEXPA11	257	27.68	8.97	-0.016	27.26	64.98
StEXPA12	256	28.72	9.87	-0.250	50.85	66.25
StEXPA13	267	28.96	8.60	-0.151	34.97	70.90
StEXPA14	247	26.46	7.52	-0.097	30.41	61.30
StEXPA15	249	26.57	9.14	-0.133	34.34	64.62
StEXPA16	263	28.68	9.48	-0.048	23.81	74.18
StEXPA17	257	27.55	9.03	-0.170	30.26	62.26
StEXPA18	269	29.48	9.26	0.052	25.30	61.71
StEXPA19	257	28.26	9.23	-0.163	25.63	68.29
StEXPA20	265	28.75	8.56	-0.075	35.62	76.98
StEXPA21	199	21.45	8.61	-0.221	30.98	72.91
StEXPA22	240	26.75	8.74	-0.123	27.71	78.33
StEXPA23	244	27.18	8.55	-0.417	27.05	65.90
StEXPA24	259	28.72	8.72	-0.179	28.42	71.51
StEXPB1	262	28.64	9.87	-0.066	30.71	82.67
StEXPB2	279	30.28	8.74	-0.092	37.47	75.13
StEXPB3	267	28.95	8.76	-0.052	39.48	71.20
StEXPB4	257	27.23	8.48	0.018	29.05	74.05
StEXPB5	256	27.61	5.35	-0.134	30.34	73.48
StEXLA1	260	28.33	8.39	0.021	29.87	79.88
StEXLB1	253	28.25	5.96	-0.108	18.96	77.83
StEXLB2	251	27.97	8.47	-0.230	17.79	78.09
StEXLB3	255	27.91	4.68	-0.248	40.85	73.80
StEXLB4	253	27.76	4.87	-0.206	35.28	80.20
StEXLB5	251	27.47	6.42	-0.139	38.23	78.84
StEXLB6	248	27.20	6.88	-0.229	27.75	76.61

### Phylogenetic analysis of expansins

Phylogenetic tree was constructed from 36 potato StEXPs, 36 *Arabidopsis* AtEXPs, 36 poplar PtEXPs, and 58 rice OsEXPs. These expansins were grouped into four clades (EXPA, EXPB, EXLA, and EXLB) based on species (Fig 1), indicating that expansins were highly conserved among species (Fig 1). The sequence similarities among EXPB, EXLA, and EXLB were more than that between them and EXPA, so EXLA and EXLB could be considered as a part of EXPB clade. In addition, the phylogenetic analysis showed expansins were most likely present before the differentiation of monocotyledon and dicotyledon, suggesting that expansins were evolved from the same ancestor (Fig 1).

**Fig 2:**
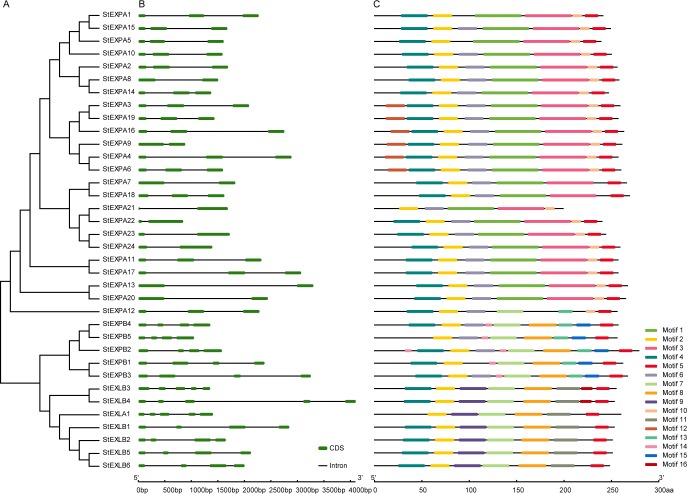
Phylogenetic relationships and structure of potato expansin genes (*StEXPs*). **a**, phylogenetic relationship. **b**, exon and intron are indicated by green box and black line, respectively. **c**, Motif, predicted by MEME online tool. A total of 16 kinds of motifs were found in the 36 StEXPs (e-value<0.05). Each gene contained 6–10 motifs, and the *X*-axis indicated the predicted amino acid no.

The potato expansin phylogenetic tree divided 36 StEXP proteins into five clusters. All the StEXPB, StEXLA, and StEXLB proteins formed into one clade, while the 24 StEXPA proteins were divided into four clades, and one of them contained 19 proteins (Fig 2A).

### Gene structure of StEXPs

Each of these 36 *StEXPs* contained 1–4 introns (Fig 2B). Specifically, *StEXLA1*, *StEXLB3*, and *StEXLB4* each contained 4 introns, *StEXPs*, *StEXPB*, *StEXLB1*, *StEXLB2*, *StEXLB5*, and *StEXLB6* each contained 3 introns, while the others each contained 1 or 2 intron(s). Among the 24 *StEXAs*, nine of them (*StEXPA7*, *StEXPA8*, *StEXPA9*, *StEXPA13*, *StEXPA20*, *StEXPA21*, *StEXPA22*, *StEXPA23*, and *StEXPA24*) each contained one intron, while the rest each had two introns. The genes within one subfamily were the same type due to they have similar length and similar intron, exons and motif structures (Fig 2B and 2C).

MEME analysis revealed that genes in the subfamilies of *StEXPA*, *StEXPB* and *StEXL* (*StEXLA* and S*tEXLB*) had common motif and unique motifs. For example, each of the StEXPA3, StEXPA4, StEXPA6, StEXPA9, StEXPA16 and StEXPA19 had an additional motif (Motif 12) at N-terminals compared with the other StEXPAs. Comparing the members of StEXPBs, StEXPB5 lacked Motif 4, StEXPB2 had an additional motif (Motif 14) at N-terminal, and StEXLB3 and StEXLB4 each had an additional motif (Motif 16) at C-terminal (Fig 2C, S1 Fig).

### Chromosomal distribution of *StEXPs*

**Fig 3:**
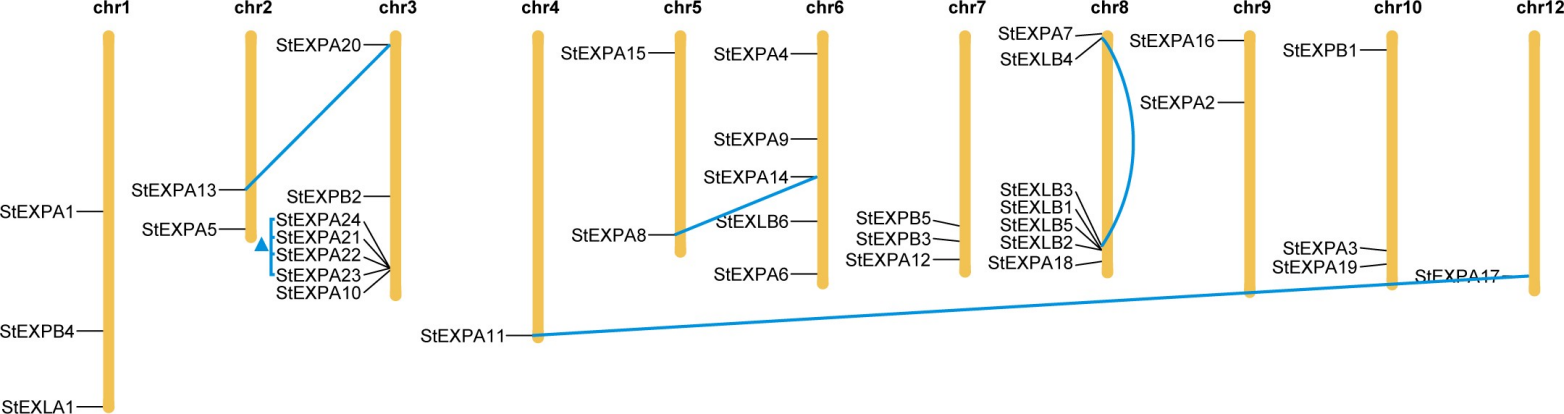
The chromosome positions of potato expansin genes (StEXP). The genes at two ends of blue line mean the potential partial duplicated expansin gene pairs (StEXPA8 and StEXPA14, StEXPA11 and StEXPA17, StEXPA13 and StEXPA20, and StEXLB3 and StEXLB4). The blue triangle indicates the four tandem repeated genes (StEXPA21, StEXPA22, StEXPA23 and StEXPA24).

The 36 *StEXP* genes were distributed on 11 of 12 chromosomes (chr. 1- chr. 10 and chr.12) of potato genome. chr. 3 and chr. 8 each contained the most seven *StEXPs*. Six *StEXPAs* and 1 *StEXPB* were located on chr.3, and 2 *StEXPAs* and 5 *StEXLBs* were present on chr. 8. In comparison, only one StEXPA was present on chr. 4 and chr. 12 (Fig 3). The 5 StEXPAs (StEXPA10 and StEXPA 21- StEXPA24) were located within a 29.0-kb region on chr. 3. And the 4 genes of StEXPA21-StEXPA24 were closely adjacent and their sequence similarity were more than 75%. Moreover, they were clustered together in phylogenetic tree. The four StEXLB genes on chr.8 (StEXLB1-StEXLB3 and StEXLB5) were located within a short region, had higher sequence similarity, and were clustered together in phylogenetic tree. The closely linked genes on chr. 3 or chr. 8 might be tandem repeated genes (Fig 3).

Moreover, among the 36 *StEXP* genes, there were four paralogous pairs, StEXPA8-StEXPA14, StEXPA11-StEXPA17, StEXPA13-StEXPA20, and StEXLB3-StEXLB4, that were dispersed segmental duplications.

### Tissue-preferential expression of potato expansin

**Fig 4:**
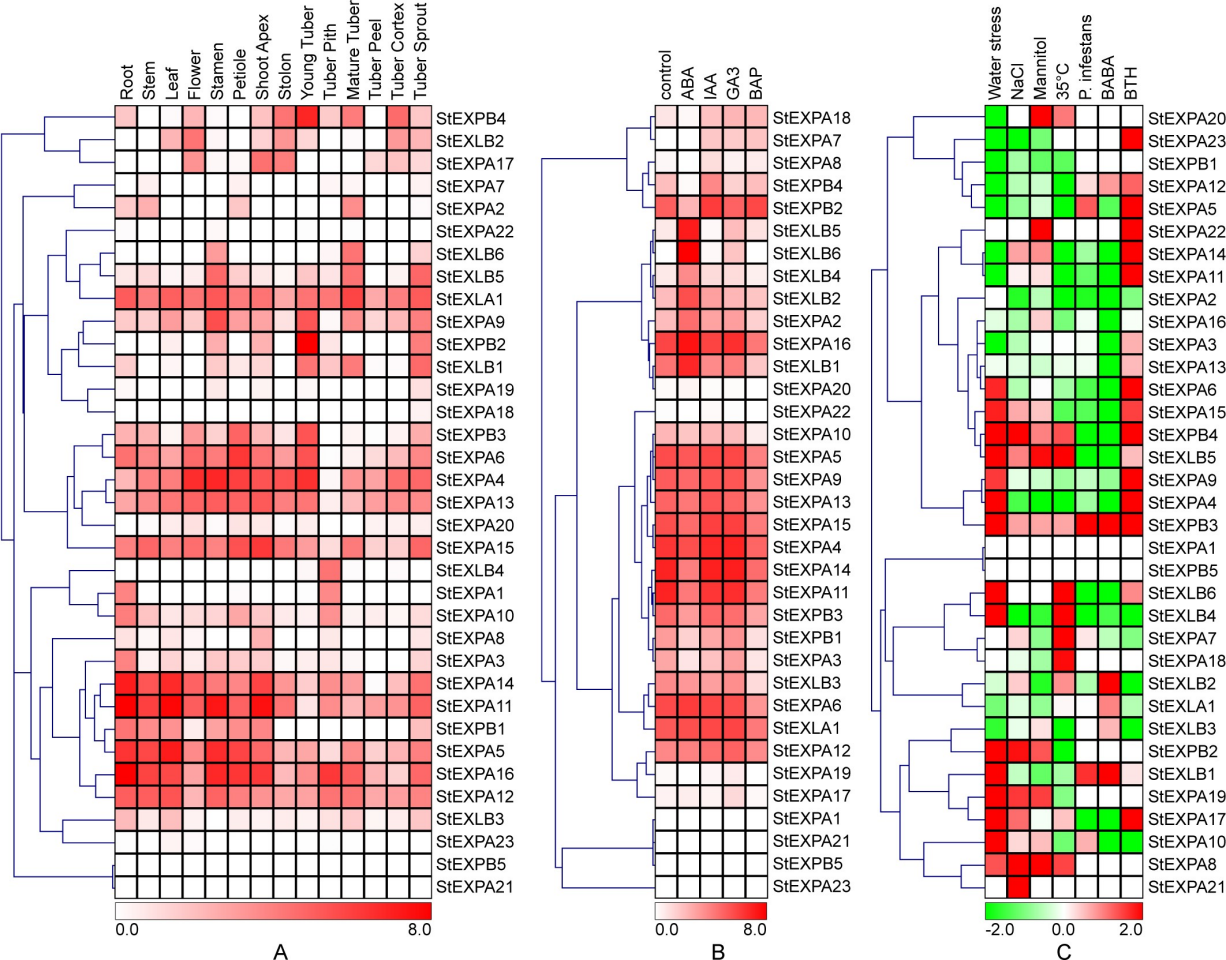
Expression profiles of potato expansin genes (*StEXP*). **a**, the expression patterns of *StEXPs* in the tissues of heterozygous diploid *Solanum tuberosum*, RH89-039-16. **b**, the expression patterns of *StEXPs* in doubled monoploid *S*. *tuberosum* Group Phureja clone, DM1-3. The whole plant *in vitro* was respectively treated for 24 hours by 50 μmol L^-1^ ABA, 10 μmol L^-1^ IAA, 50 μmol L^-1^ GA3, and 10 μmol L^-1^ BAP. **c**. the expression patterns of *StEXPs* in DM1-3. The whole plant *in vitro* was exposured to 150 mmol L^-1^ NaCl, 260 μmol L^-1^ Mannitol, 35°C high temperature, respectively, or three separate detached leaves of the plant was spray inoculated by *Phytophthora infestans*, BABA and BTH, respectively [33]. All FPKM = 0 of transcripts were replaced by FPKM = 0.01, and the FPKM data in Fig 4A and 4B was undergone a log_2_ (FPKM_Treatment_ / FPKM_Control_) transformation.

The gene members of *StEXPs* showed significantly different expression levels. *StEXPB2* transcript was the most abundant among *StEXPs*. It had the FPKM value of 852.8 in young tuber, while was absent in root, stem, flower, and other tissues (Fig 4A). This suggested that *StEXPB2* played an important role during tuber development. *StEXPA11*, *STEXPA16*, *StEXPA4*, *StEXPA14*, and *StEXLA1* transcripts also showed relatively high abundance in most tissues and their average FPKM values were 76.3, 56.3, 38.9, 32.56 and 23.65, respectively. However, *StEXPA21* and *StEXPB5* transcripts were absent in all tissues. Different *StEXP* genes are expressed differently among tissues. The average FPKM values of all *StEXPs* were 28.5 in roots and 20.0 in leaves, while it was only 1.3 in tuber peel.

### Differential expression of *StEXP* after phytohormone treatment

Thirty-one of 36 *StEXP* genes responded to ABA, IAA, GA3, and BAP induction in different ways (Fig 4B, Table 2). Among them, there were 8, 7, 8, and 3 *StEXP* genes showed up-regulation under ABA, IAA, GA3, and BAP treatments, respectively (Fig 4B, Table 2). And all the *StEXLB* DEGs induced by ABA and GA3 were up-regulated. Specially, *StEXPA7* and *StEXLB6* were remarkably up-regulated by several hormones. *StEXPA7* and *StEXPA18* were up-regulated by the three types of hormone (IAA, GA3 and BAP). Besides, five StEXP genes (*StEXPA2*, *StEXPA8*, *StEXLB2*, *StEXLB5* and *StEXLB6*) were up-regulated by two of the four hormones (ABA, IAA, GA3 and BAP), and another 11 StEXP genes were up-regulated by one hormones. These results not only show the different expression patterns of potato expansin gene in response to different hormones but also reveal similar functions within the same expansin gene group.

**Table 2:** The expression levels of potato expansin genes (*StEXPs*) under hormone and stress treatments.

Gene	ABA	IAA	GA3	BAP	Salt	Mannitol	35°C	Water stress	*P*. *infestans*	BABA	BTH
StEXPA1											
StEXPA2	2.77		1.14		-1.70				-1.74	-10.19	
StEXPA3	-2.84	-1.06		-2.96				-1.99			
StEXPA4				-1.55	-1.41	-2.64	-2.34	2.19		-2.34	4.23
StEXPA5				-1.79			-3.76	-3.25	1.28	-1.31	3.11
StEXPA6				-2.49				1.71	-1.38		2.03
StEXPA7		11.23	11.12	11.41			3.08				
StEXPA8		2.57		1.51	3.98	2.57	1.47	1.35			
StEXPA9				-1.83				1.56			3.41
StEXPA10				-3.04			-1.12	2.14			
StEXPA11	-2.80			-2.85			-4.08	-2.44	-1.21	-5.53	2.70
StEXPA12			1.26				-2.59	-2.82			1.22
StEXPA13				-1.52						-2.59	
StEXPA14	-2.95			-2.36			-3.25	-2.56		-3.47	2.43
StEXPA15				-1.45			-1.34	1.76	-1.33	-4.25	1.50
StEXPA16	1.62			-1.66			-1.05			-4.03	
StEXPA17			1.91	-1.07	1.15			11.89			2.36
StEXPA18	-2.09	1.84	2.39	2.25			1.93				
StEXPA19			2.07		1.57	1.51		7.47			
StEXPA20	1.08						1.05	-2.81			
StEXPA21											
StEXPA22		1.34									
StEXPA23											
StEXPA24											
StEXPB1	-2.14			-2.87		-1.21	-1.27	-3.31			
StEXPB2	-3.01	1.11			1.88	1.34	-10.38	4.77			
StEXPB3	-1.70			-2.02				5.32			
StEXPB4	-4.61	2.02			2.20		1.37	5.83	-1.97		3.58
StEXPB5											
StEXLA1				-1.04				-1.07			
StEXLB1	2.49			-3.04		-1.21		6.75	1.63	2.54	
StEXLB2	3.77	1.07				-1.72				2.92	-2.99
StEXLB3				-3.20			-4.32	-1.60			-2.80
StEXLB4	4.21				-2.02	-1.59			-2.55	-1.43	-5.25
StEXLB5	7.86	-1.83	2.44			1.92	3.54	7.90	-3.34	-14.33	
StEXLB6	17.98		11.41				13.90	14.70	-2.03	-9.05	
	8(8)	7(2)	8(0)	3(17)	5(3)	4(5)	7(11)	14(9)	2(8)	2(11)	10(3)

**Note:** The data is the log_2_(Fold change) >1. The number in front of and in bracket are the gene No. of up- and down-regulated, respectively.

### Induced expression of *StEXP* exposure to biotic and abiotic stresses

Most of the identified *StEXP* genes were up- or down-regulated when exposed to different biotic and abiotic stresses (Fig 4C, Table 2). Specifically, *StEXPs* responded to NaCl and mannitol treatments similarly. The number of differentially expressed genes (Log2 fold change >1) under NaCl and mannitol treatments was the same, with eight genes were up-regulated and nine were down-regulated. And *StEXPA8*, *StEXPA19*, *StEXPB2* were up-regulated, while *StEXPA4* and *StEXLB4* were down-regulated under both treatments. There were 23 *StEXP* genes in response to water stress, with 14 of them being up-regulated and 9 of them down-regulated. Among the up-regulated genes, *StEXPA4*, *StEXPA15*, *StEXLB1*, *StEXLB5* and *StEXLB6* showed 20-fold more transcript abundance than the control, and among the down-regulation genes, the transcription levels of *StEXPA5*, *StEXPA11*, *StEXPA 12*, and *StEXPA14* were decreased by nearly 95%. The expression levels of 18 genes were changed under high temperature stress, and seven of them (*StEXPA7*, *StEXPA8*, *StEXPA18*, *StEXPA20*, *StEXPB4*, *StEXLB5* and *StEXLB6*) were up-regulated. *StEXLB6* showed the highest expression levels under both drought and high temperature stresses, and its transcription levels under the two stresses were similar. While *StEXPB2* was down-regulated the most by high temperature stress.

The effects of *P*. *infestans* and disease resistant inducer BABA on *StEXP* genes were very similar, but the effect was significantly different from that of BTH. Gene expression patterns (Fig 4C) showed that 14 *StEXP* genes were transcribed in similar ways when they were induced by *P*. *infestans* or BABA, whereas 10 of them were transcribed in an opposite way when induced by BTH (Fig 4C, Table 2).

In summary, most of the *StEXPs* showed more complex expression patterns in response to biotic and abiotic stresses than to hormones. Five genes (*StEXPA1*, *StEXPA21*, *StEXPA23*, *StEXPA24* and *StEXPB5*) did not show significant transcription changes under either biotic and abiotic stresses or hormones. It was likely due to they had low expression level in tissues, because a small number of reads were detected from RNA-Seq data.

### Weighted gene co-expression network analysis (WGCNA) of *StEXPs*

**Fig 5:**
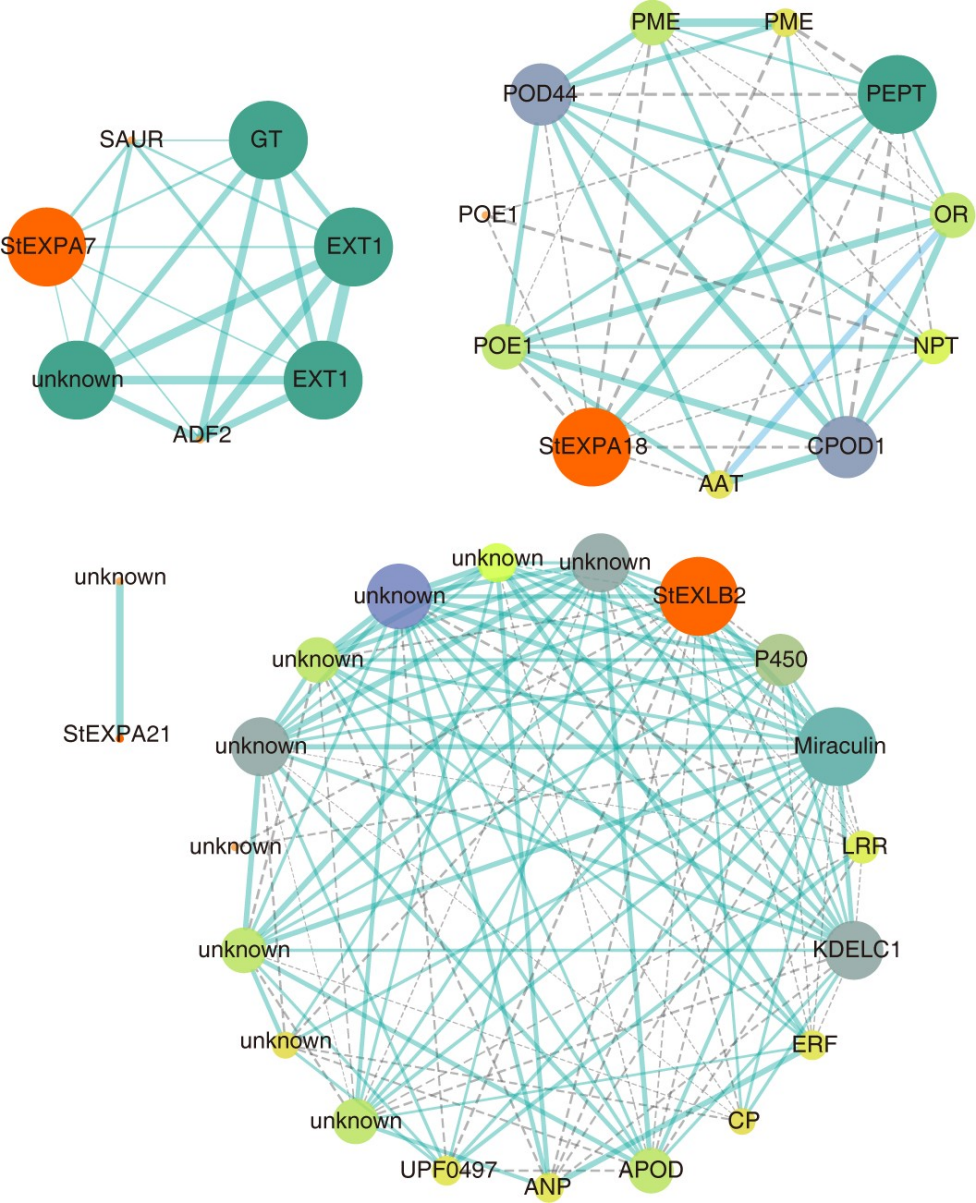
The co-expression network of potato expansin genes (StEXP). The dash or solid lines indicate weight value of edges between 0.85 and 0.90 or >0.90.

In the WGCNA, four *StEXPs* (*StEXPA7*, *StEXPA18*, *StEXPA21* and *StEXLB2*) were found to be involved in the co-expression networks with other genes (Fig 5, S2 Fig). Specifically, *StEXPA7* and *StEXPA18* were involved in the same co-expression network and interacted with 409 genes. The directly adjacent genes of *StEXPA7* were mainly associated with the development of cell wall and the formation of cytoskeleton. And the genes directly adjacent to *StEXPA18* were involved in cell wall development, nutrient uptake and transport, and stress resistance. *StEXPA21* was co-expressed only with a gene with unknown function. StEXLB2 and other 289 genes constituted a co-expression network. In this network, StEXLB2 was directly neighboring 18 genes, half of which had unknown functions and the other half were related to biotic and abiotic resistances (Table 3).

**Table 3:** Function of protein involved in co-expression network.

Label	Annotation	Function	Reference
SAUR	SAUR^a^ family protein	Regulate plant growth and development, promote cell expansion.	[39]
GT	Glycosyltransferase	Involved in the biosyntheses of cell-wall polysaccharides.	[40]
EXT1	Extensin Ext1	Involved in building and maintaining the growing primary cell wall.	[41]
ADF2	Pollen specific actin-depolymerizing factor 2	Reorganizing the actin cytoskeleton.	[42]
POE1	Pollen ole e 1 allergen and extensin family protein	Developmental regulators in plant tissues.	[43]
POD44	Peroxidase 44	Tolerant stress, biosynthesis and degradation of lignin in cell walls, auxin catabolism, etc.	[44]
PME	Pectinesterase	Involved in cell wall stiffening.	[45]
PEPT	Oligopeptide transporter	Involve in amino acids, nitrogen, or carbon transport.	[46]
OR	Oxidoreductase	Redox activity.	[47]
NPT	Inorganic phosphate transporter	Acquisition of Phosphorus in roots.	[48]
CPOD1	Cationic peroxidase 1	Biotic and abiotic stress.	[49]
AAT	Anthocyanin acyltransferase	Anthocyanin synthesis.	[50]
P450	Cytochrome P450		
Miraculin	Miraculin		
LRR	Leucine-rich repeat protein	Biotic and abiotic stress.	[51,52]
KDELC1	KDEL^b^ motif-containing protein 1	Endoplasmic reticulum retention.	[53]
ERF	Ethylene-responsive transcription factor	Biotic and abiotic stress.	[54]
CP	Cysteine protease	Biotic and abiotic stress.	[55,56]
APOD	Suberization-associated anionic peroxidase	Involved in suberization of tuber development.	[57]
ANP	Anthranilate N-benzoyltransferase protein	Disease resistance.	[58]
UPF0497	UPF^c^0497 membrane protein	Response to abiotic stress.	[59]
unknown	Gene of unknown function		

^a^SAUR Small auxin-up RNAs.

^b^KDEL motif: Lys-Asp-Glu-Leu.

^c^UPF uncharacterized protein family.

### Expression patterns of *StEXLs* and co-expression network involved genes under abiotic stresses as determined by qRT-PCR

**Fig 6:**
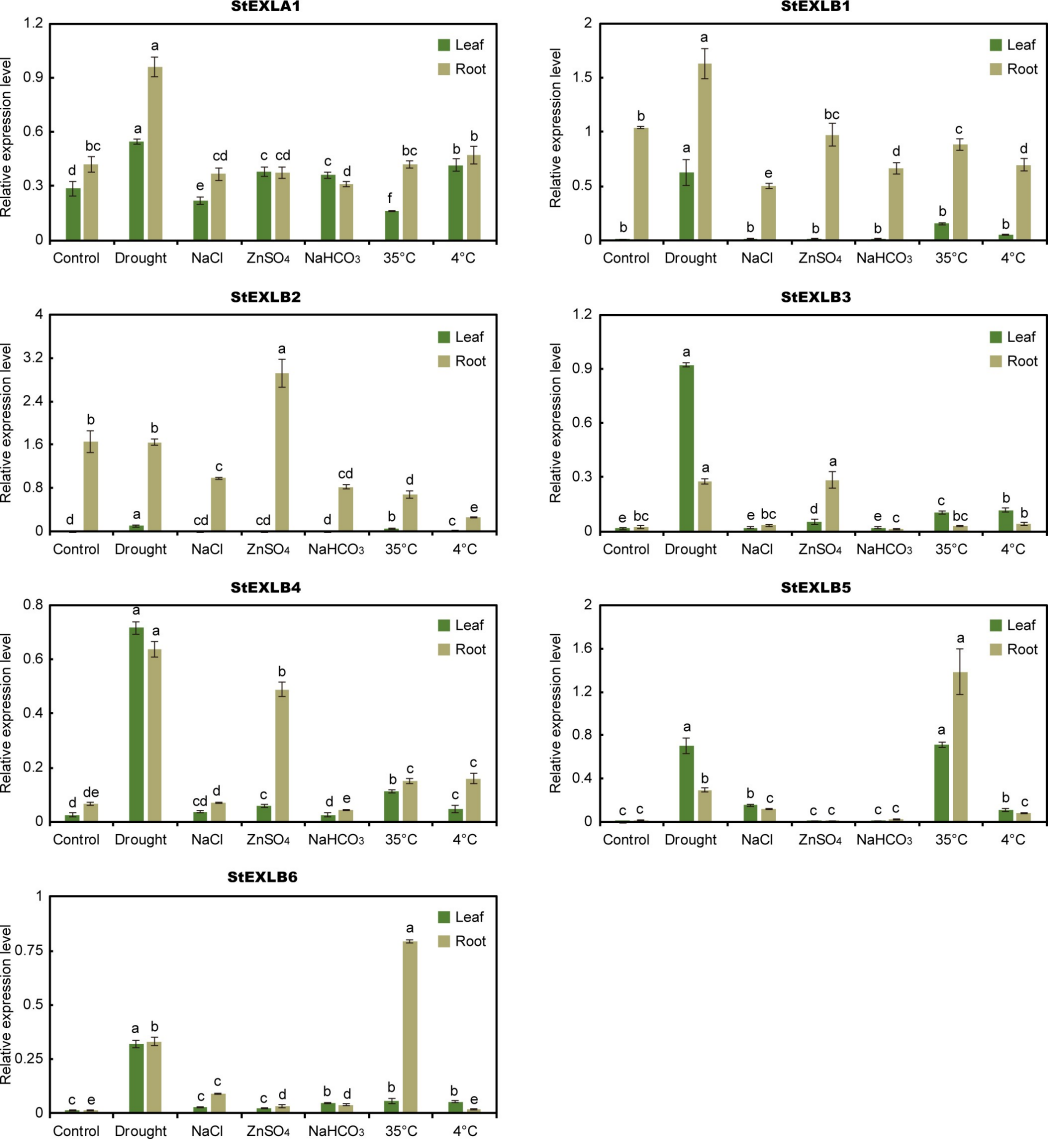
Expression profiles of potato expansin-like genes under various abiotic stresses. Values represent mean± standard deviation of three replicates. Different letters indicate significant differences by Tukey's test (P < 0.05) using PROC ANOVA in SAS 9.4.

Our analysis above indicated that *StEXLB* genes contributed to the resistances of a wide range of abiotic stresses. qRT-PCR results (Fig 6) confirmed that six *StEXLBs* (*StEXLB1*, *StEXLB3*, *StEXLB4*, *StEXLB5*, and *StEXLB*6) and *StEXLA1*, were significantly up-regulated in roots and leaves under drought stress. And among the seven up-regulated genes, the transcription levels of *StEXLB3*, *StEXLB4*, *StEXLB5* and *StEXLB6* in leaves changed the most, which were 56.0, 28.4, 70.1 and 21.2 folds higher than that of control, respectively. *StEXLB1-6* genes were up-regulated under the heat treatment, in which, the *StEXLB3*, *StEXLB4*, *StEXLB5* and *StEXLB6* transcription levels in roots were the highest four, which were 11.7, 9.6, 94.3 and 56.4 folds greater than that of control, respectively. The genes *StEXLB2-StEXLB4* were up-regulated under the ZnSO_4_ stress and their transcription levels were significantly increased in roots. And among them, *StEXLB4* were up-regulated the most, with 6.4 folds greater of that in control. Although the four genes (*StEXLB3-StEXLB6*) showed mild expression level under NaCl, NaHCO_3_ and cold treatments, they were involved in a wide range of plant resistance.

**Fig 7:**
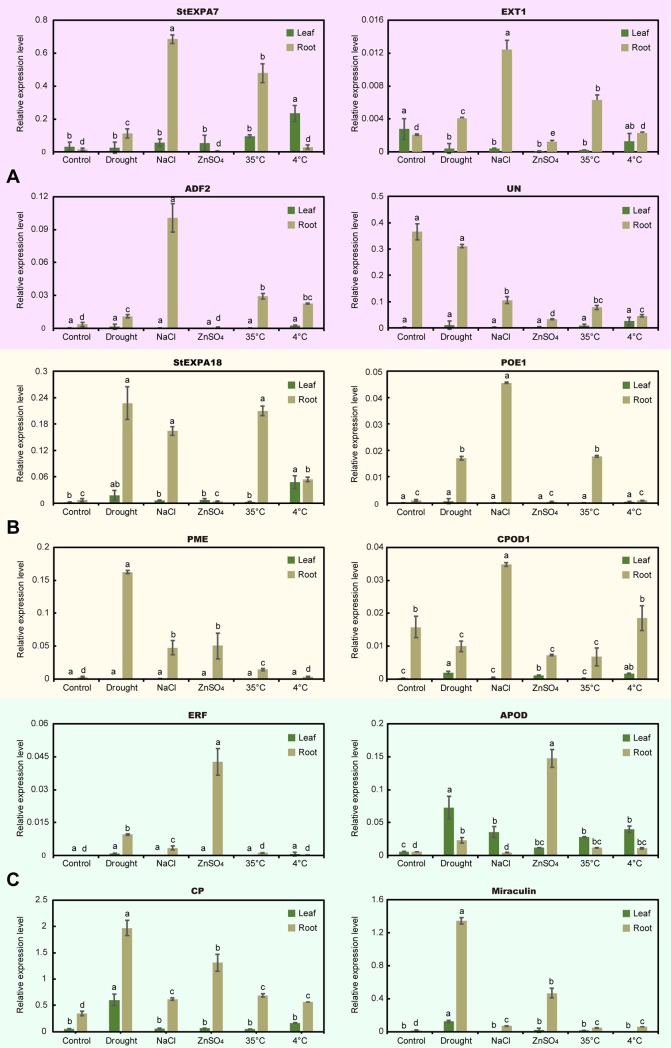
Expression profiles of co-expression network involved genes under various abiotic stresses. a, StEXPA7 and 3 of its directly adjacent genes. b, StEXPA18 and its directly adjacent genes. c, 4 directly adjacent genes to StEXLB2. Values represent mean± standard deviation of three replicates. Different letters indicate significant differences by Tukey's test (P < 0.05) using PROC ANOVA in SAS 9.4.

The qRT-PCR analyses of 4–5 genes within the co-expression network of *StEXPA7*, *StEXPA18* and *StEXLB2* were also be performed. *StEXPA7* and *StEXPA18* which were co-expressed in a same network (S2 Fig) showed similar expression patterns. They both were significantly induced under drought, NaCl and heat stresses in root, and cold induced in leaf (Fig 7). In the co-expression network of *StEXA7*, 3 direct adjacent genes were analysis by qRT-PCR, of them, EXT1 (PGSC0003DMG400011599) and ADF2 (PGSC0003DMG400029916) were similar to *StEXPA7* (Fig 7), with up-regulation under drought, NaCl and heat stresses in root. And the expression of them were significantly correlated (Table 4). In the co-expression network of *StEXPA18*, POE1 (PGSC0003DMG400030033) and PME (PGSC0003DMG400018037) were significantly correlated to *StEXPA18* (Table 5). The most obvious response of *StEXLB2* was the up-regulation under ZnSO_4_ treatment in root (Fig 6). ERF (PGSC0003DMG400013401), APOD (PGSC0003DMG400022342), CP (PGSC0003DMG400008004) and miraculin (PGSC0003DMG400015219), these *StEXLB2* co-expressed genes also exhibited a response to ZnSO_4_ (Fig 7). The abiotic responsive correlations of ERF, APOD and CP to StEXLB2 were significantly (Table 6).

**Table 4:** Pearson's correlation coefficient of StEXPA7, EXT1, ADF2 and an unknown function gene.

	StEXPA7	EXT1	ADF2
EXT1	0.889**		
ADF2	0.871**	0.945**	
UN	0.028	0.257	0.113

Pearson's correlation coefficients were calculated using PROC CORR of SAS 9.4.

"*" indicates P<0.05

"**"indicates P<0.01.

**Table 5:** Pearson's correlation coefficient of StEXPA18, POE1, PME and CPOD1.

	StEXPA18	POE1	PME
POE1	0.759**		
PME	0.679*	0.450	
CPOD1	0.456	0.772**	0.282

Pearson's correlation coefficients were calculated using PROC CORR of SAS 9.4.

"*" indicates P<0.05

"**"indicates P<0.01.

**Table 6:** Pearson's correlation coefficient of StEXLB2, ERF, APOD, CP and miraculin.

	StEXLB2	ERF	APOD	CP
ERF	0.947**			
APOD	0.740**	0.851**		
CP	0.621*	0.594*	0.369	
Miraculin	0.463	0.434	0.226	0.910**

Pearson's correlation coefficients were calculated using PROC CORR of SAS 9.4.

"*" indicates P<0.05

"**"indicates P<0.01.

## Discussion

Expansins have been recently found in many plant species. For example, there were 52 expansins (36 *EXPAs*, 6 *EXPBs*, 3 *EXLAs*, and 7 *EXLBs*) identified in tobacco [7]. In tomato, 38 expansins were found, which include 25 *EXPAs*, 8 *EXPBs*, 1 *EXLA*, and 4 *EXLBs* [8]. In this study, we identified a total of 36 potato expansins, including 24 *EXPAs*, 5 *EXPBs*, 1 *EXLA*, and 6 *EXLBs*. The difference in gene copies in expansin family and subfamily among species is likely due to biological evolution resulting from varied requirements in growth and development of plant and environmental adaptation [8]. In addition, the varied motif structures among different subfamilies of expansins indicate their possible differences in action and function. For example, of the 11 cadmium-responded differential expression expansins in *P*. *americana*, EXPA was down-regulated while *EXPB* was up-regulated [27]. In potato, all *StEXPBs* were differential expression under ABA treatment (Fig 4B). Whether the genes in one subfamily show similar functions in potato need to be validated.

Gene expression pattern can provide insights into gene function. That expansins were involved in root or root hair development and stress tolerance have been reported in many species, such as *A*. *thaliana* [60,61], grapevine [62], and Tibetan wild barley [63]. The potato expansin genes, such as *StEXPA5*, *StEXPA11*, *StEXPA14*, and *StEXPA16*, had higher expression levels in root, leaf and stem than in other tissues, indicated that they might take effects in plant development. They also expressed in high levels under IAA and GA3 treatments. In Jung’s report [29], these 4 expansin genes were involved in tuber development and etiolated stem elongation, and also be induced in varying degrees under IAA treatment. Expansin genes also participated in the development of tuber in some species, such as *Rehmannia glutinosa*, *Smallanthus sonchifolius* [64,65]. Simultaneously, expansins are pleiotropic and play multiple roles during plant growth and development as well as stress resistance. For example, the overexpression of *TaEXPA2* and *TaEXPB23* from wheat not only contributed to the drought resistance ability of transgenic tobacco, but also increased its seed number, and *TaEXPB23* was also involved in leaf area development and internode length [16]. Many potato *StEXPs* were found to be involved in plant growth and stress resistance too. Most of the adjacent genes of *StEXPA7* or *StEXPA18* in their co-expression network were related with the development of cell wall (Fig 5, Table 3), and *StEXPA7* and *StEXPA18* could also be induced by abiotic stresses (Fig 7). In comparison with *StEXPA7* and *StEXPA18*, *StEXLB2* was associated with more biotic and abiotic stresses related genes in the co-expression network (Fig 5, Table 3). The qRT-PCR analysis and the co-expression network deduced by the expression in diploid potato were showed a similar correlation implied potato expansins act in common modes among different genotypes.

ABA is a stress signal [66], which could up-regulate eight potato expansin genes. Of these eight genes, there were 5 *StEXLBs* (Table 2). *StEXLB4*, *StEXLB5*, and *StEXLB6* showed changed expression levels under ABA, high temperature, and water stresses (Fig 4B and 4C; Fig 5), indicated that they also work in a wide range of abiotic resistances. In addition, it had been reported that the overexpression of *PtEXPA8* in *Populus tomentosa* and *AstEXPA1* in *Agrostis stolonifera* enhanced the transgenic plants tolerance to many stresses [67,68]. It also indicates that expansin has potential of resistance to wide abiotic stresses. The qRT-PCR confirmed the above results, all the 6 *StEXLB* genes could be induced by one or more stress treatments. In tomato, the closely related species of potato, there were three of four *SlEXLB* genes inducible by stress treatments [8]. More specificity of the pleiotropic roles of *EXLB* in tolerance to abiotic stresses. Furthermore, *StEXLBs* were mainly distributed on chromosome 8 (Fig 3), and *StEXLB3* and *StEXLB4* were the potential duplicated gene pairs, suggesting a selective advantage exists for retaining these gene copies [69]. Therefore, we speculate that the *EXLB* subfamily in potato may also play important roles in plant adaptability [69,70].

The expansin genes can loosen cell walls, and the loosened cell walls can lead to vulnerable cells that are easy to be damaged by biotic invaders [71]. We predicted that the up-regulations of *StEXPA5*, *StEXPB3*, and *StEXLB1* were likely to increase cell wall loosening, thus increase the chance of *P*. *infestans* invasion. The down-regulations of *StEXPA2*, *StEXPA6*, *StEXPA11*, *StEXPA15*, *StEXPB4*, *StEXLB4*, *StEXLB5*, and *StEXLB6* were likely to improve the potato resistance to disease. The induction mechanisms of disease resistance inducers BTH and BABA are different [72], which could be indicated by the different responsible patterns of *StEXP* genes. The inducers can work much efficiently only when the induction of disease resistance by inducers is similar to the way that plant responses. The way that *StEXPs* responded to *P*. *infestans* is the same as that of BABA induction, therefore BABA likely induced the resistance to *P*. *infestans* in potato through activating expansins.

## Conclusions

In this study, 36 putative expansin genes in potato were identified and analyzed. The *StEXP* gene family was divided into four groups based on phylogenetic analysis, indicating that *StEXP* genes showed a high level of functional divergence. *StEXP* genes exhibited tissue-specific expression patterns and distinctly modulated by exogenous hormones, biotic or abiotic stress conditions. The preferential expression of *StEXPB2* in young tubers indicated its role in tuber development. Many of the *StEXP* genes, especially the *StEXLB* subfamily members, were significantly up-regulated under water stress, high temperature, and other abiotic stress conditions. The tissue-specific expression patterns of expansin genes would provide insights for their functional characterization in potato. These resultswere valuable for understanding the biological functions of expansins during the growth and development of potato, especially tuber development.

## Supporting information

S1 Fig Motifs of potato expansins. (TIF)

S2 Fig The co-expression network involved in potato expansins genes (*StEXPs*). (TIF)

S1 Table Primers for qRT-PCR analysis. (DOCX)
