# Supplementary figures and images for: A comprehensive expression analysis of the expansin gene family in potato (Solanum tuberosum) discloses stress-responsive expansin-like B genes for drought and heat tolerances
Source: PLoS One. 2019 Jul 18;14(7):e0219837. doi: 10.1371/journal.pone.0219837 (PMC6638956; doi:10.1371/journal.pone.0219837)

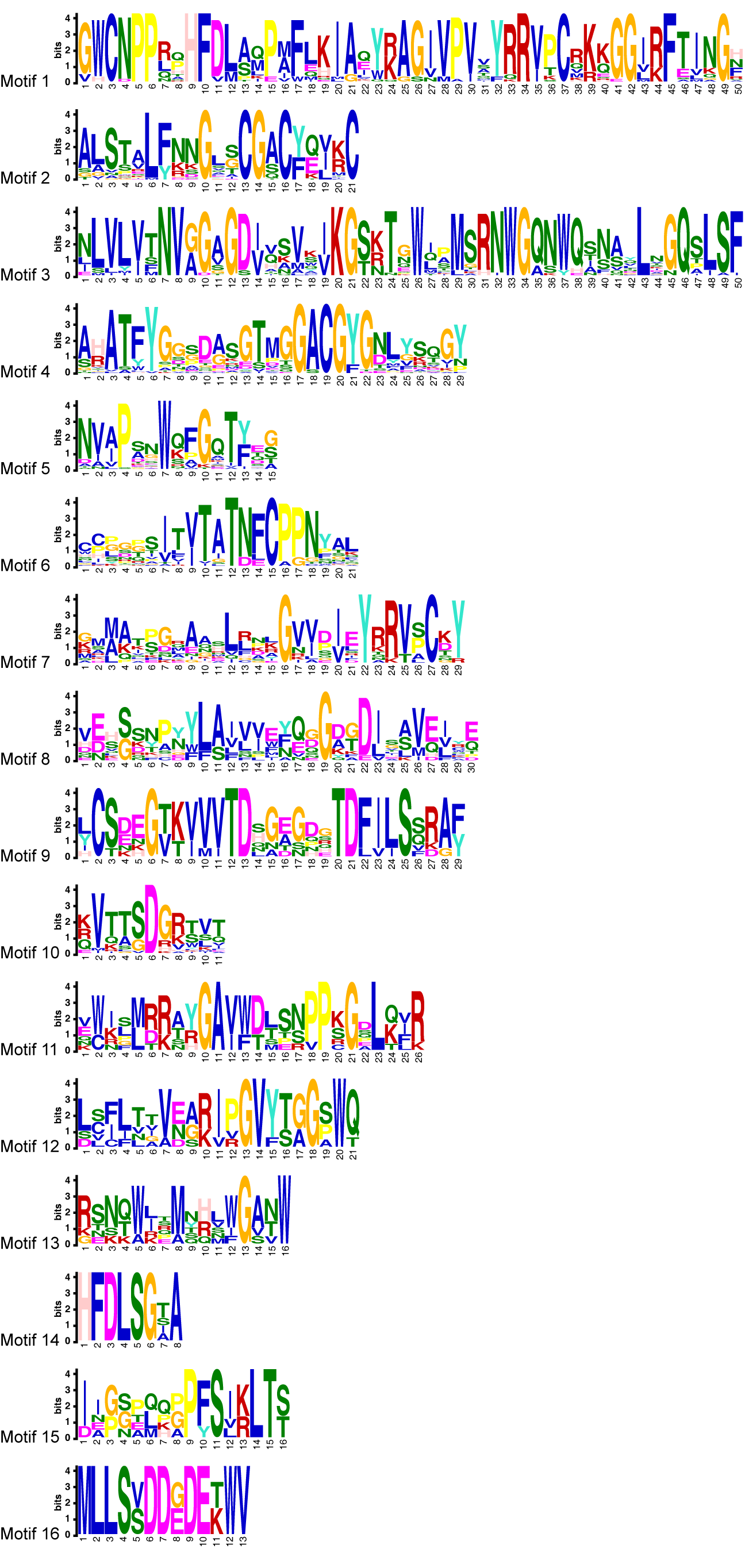

Supplement: S1 Fig — (TIF) [file pone.0219837.s001.tif]

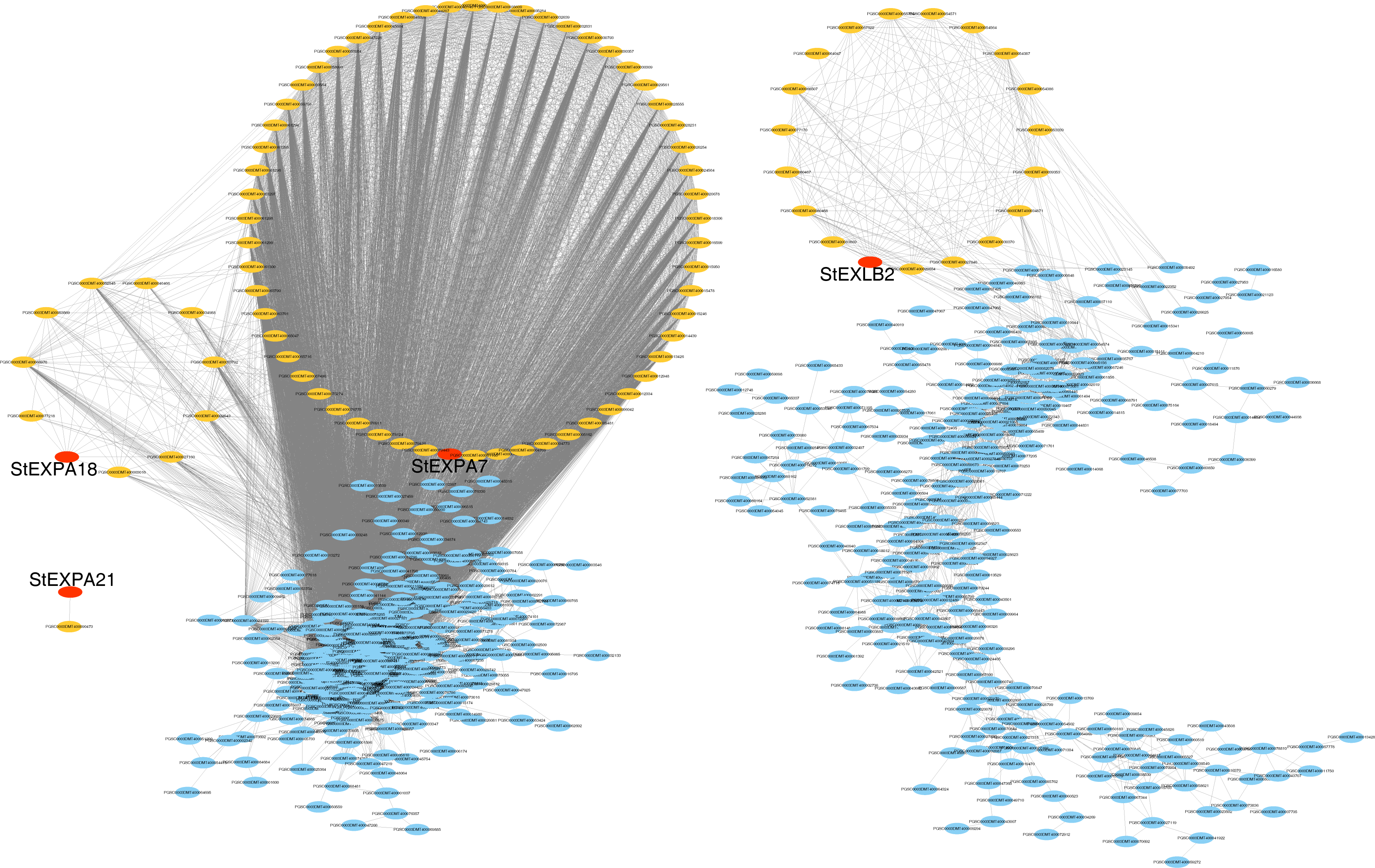

Supplement: S2 Fig — (TIF) [file pone.0219837.s002.tif]
