# Supplementary material for: A comprehensive expression analysis of the expansin gene family in potato (Solanum tuberosum) discloses stress-responsive expansin-like B genes for drought and heat tolerances
Source: PLoS One. 2019 Jul 18;14(7):e0219837. doi: 10.1371/journal.pone.0219837 (PMC6638956; doi:10.1371/journal.pone.0219837)
Supplement: S1 Table — (DOCX) [file pone.0219837.s003.docx]

**S1 Table. Primers for qRT-PCR analysis.**

| **Gene** | **Forward primer** | **Reverse primer** |
| --- | --- | --- |
| StEF1α | CTGCACTGTGATTGATGCCCCTGGT | CTTCGGGGTGGTAGCATCCATCTTGT |
| StEXLA1 | GTGCTGGATGTGGTGCTTGTTATCAGA | CTTGCCTTTGTAATCACAAGGAACTCTT |
| StEXLB1 | CCCTGATTGCTATGGAACCCCTAGTG | TGGTCACCTTCACCATGATCTGTTAC |
| StEXLB2 | CTCTGATGGCAAAGGAACACCTACTG | CCTCACCACTATCAGTCACCACCAC |
| StEXLB3 | AGGAACAGAAACTGGGAGTTGTGGA | AGTCTGTGCGATCACCTGCACCTTG |
| StEXLB4 | TGGAGCAACAATCAACGGTGGAGATG | GCAGCAGCATCTTTTGTTTGGGCCAT |
| StEXLB5 | CAGCCAAACCTTAGTTTCCAAGGCATC | GCATTGTTCTTGCACCTAACCTGGT |
| StEXLB6 | CCAGATGGCATGGGGACACCAAGTG | CACAACTACTTTTGTGCCCTCATCAC |
| StEXPA7 | GTCCCTGTTTCTTATCGCAGGGTAC | GAAGCTCCCCAATTATGGCTCATAGC |
| EXT1 | GCCACATGCACTATGGCTTATTCAC | CCTTTTCTGACTCTGAGGGTACCTTG |
| ADF2 | CGCCCAACAAGTGGTTGTGGAGAAG | ATGTGTCGGGAGACCAGGCAATGAAG |
| UN | GCCATGCCAATTCCACATCCATATTC | CAGCCCCTTTAATATTCGGACTCGTC |
| StEXPA18 | AACCTTTGTCCACCTAATTGGTCCC | CACCTTTCTTAACGCAAGGTACCCTG |
| POE1 | ATGGCAAGCAGCCAACTTGTCCTGAT | ACTAACTTTAGCTCCTGCAATGGGCT |
| PME | TTAACTCTGGCACCGTTGTGGTTAC | CGTTGTGAATGGACATATAGAGTGTCT |
| CPOD1 | CCATGATTGCTTTATCAGGGGTTGTG | CCTGGTACAGCATACTTTATACCACC |
| ERF | GGGGAAATATGCAGCCGAAATTCGCG | GAGCTCCTAGGTAAGTTATACTCCTC |
| APOD | TCGTTGGAATCAGGCGTAGCCAGTA | AGCAGAATACCTCCATCACAACCATC |
| CP | GGATGGAAGATGTGACCAAACAAGGAA | ACCATGGTCCACTGCTGCACCACA |
| Miraculin | GGTGGACTCACCATGGACAGTATTG | GCTTCCATTGTGTTGTCTGAACACAG |
